# Supplementary material for: American triatomine species occurrences: updates and novelties in the DataTri database
Source: GigaByte. 2022 May 31;2022:gigabyte62. doi: 10.46471/gigabyte.62 (PMC9930499; doi:10.46471/gigabyte.62)
Supplement: Translation

# Translation: Presencias geográficas de triatominos americanos: actualizaciones y novedades de la base de datos DataTri

**Authors:** Soledad Ceccarelli, Agustín Balsalobre, María Eugenia Vicente, Rachel Curtis-Robles, Sarah A. Hamer, José Manuel Ayala Landa, Jorge E. Rabinovich, Gerardo A. Marti

**Affiliations:** ^1^ Centro de Estudios Parasitológicos y de Vectores (CEPAVE-CCT-La Plata-CONICET-UNLP), La Plata, Buenos Aires 1900, Argentina; ^2^ Consejo Nacional de Investigaciones Científicas y Técnicas (CONICET), Buenos Aires, Argentina; ^3^ College of Veterinary Medicine and Biomedical Sciences, Texas A&M University, College Station, Texas, USA; ^4^ Museo del Instituto de Zoología Agrícola (MIZA), Maracay, Venezuela

## Abstract

El agente causal de la Enfermedad de Chagas (*Trypanosoma cruzi*) es transmitido a los mamíferos - incluyendo al ser humano - principalmente por insectos vectores de la subfamilia Triatominae (Hemiptera: Reduviidae). Actualmente, la subfamilia incluye 157 especies (154 actuales y tres extintas), agrupadas en 18 géneros y cinco tribus. Presentamos la descripción de un subconjunto de datos (7852 registros) de presencias de triatominos americanos, como una actualización de la más completa e integrada base de datos disponible hasta el momento a escala continental. Los nuevos registros georreferenciados fueron obtenidos de una revisión sistemática de referencias publicadas y datos cedidos por colegas. Los nuevos datos corresponden a 101 especies y 14 géneros de 22 países americanos en el intervalo de tiempo entre 1935 y 2022. Las novedades más importantes refieren a: (i) la incorporación de nuevas especies, (ii) sinonimias y transferencias formales de especies, y (iii) actualizaciones temporales y geográficas de registros de especies. Alentamos a la utilización de esta información, especialmente para contribuir a la vigilancia entomológica implicada en la Enfermedad de Chagas.

## Descripción de datos

### Contexto

La enfermedad de Chagas, producida por el protozoario *Trypanosoma cruzi* (Chagas, 1909) (Kinetoplastida, Trypanosomatidae), es transmitida principalmente por insectos vectores (a través de sus heces) conocidos como triatominos (Hemiptera: Reduviidae: Triatominae), aunque también puede transmitirse de madre a hijo, por transfusiones de sangre o por el trasplante de algunos órganos infectados, y por transmisión oral a través de la ingesta de alimentos y/o bebidas contaminadas. Estas múltiples vías de transmisión hacen de la enfermedad de Chagas un importante problema de salud pública, principalmente en América, pero también en otros continentes [1.]. Actualmente, la subfamilia Triatominae consta de 154 especies existentes y 3 fósiles (143 especies presentes en América y 14 especies presentes en Asia) agrupadas en cinco tribus y 18 géneros [2.]. Realizar una recopilación de información geográfica y ecológica sobre los triatominos es particularmente importante, dado que estos insectos vectores son una de las principales vías de transmisión de *T. cruzi*. Una vez disponible, dicha información podrá ser utilizada para llevar a cabo acciones y programas de apoyo para la prevención de enfermedades y el control de vectores por parte de las agencias de salud pública. Luego de la publicación sobre especies de triatominos americanos de Carcavallo *et al.* [3.], una nueva base de datos completa e integrada sobre ocurrencias de triatominos se encuentra disponible desde la publicación de DataTri [4.]. Sin embargo, dado que el número de especies se ha incrementado y ha habido varios cambios en la taxonomía de los triatominos, esta información necesita una actualización. En este contexto, el objetivo principal de este trabajo es describir las características de un subconjunto de datos sobre la presencia de triatominos americanos (de aquí en adelante, ‘Subconjunto de datos Americano’), destacando las actualizaciones e inclusiones más importantes realizadas después de cinco años de revisión de nuevas fuentes de datos. Este Subconjunto de datos Americano se integró para actualizar el conjunto de datos de ocurrencia de triatominos Americano (de aquí en adelante, ‘Nuevo conjunto de datos Americano’) disponible al día de la fecha en la plataforma “Global Biodiversity Information Facility” (GBIF) [5.], con un total de 19600 registros. A su vez, este Nuevo conjunto de datos Americano se complementa con el conjunto de datos sobre las especies de triatominos presentes en Argentina (de aquí en adelante, “Conjunto de datos Argentino”), también almacenado en GBIF (Figure 1). Este trabajo es el resultado de una revisión exhaustiva de información pública combinada con una importante colaboración interinstitucional, que integró datos no solo geográficos sino también ecológicos para todas las especies de triatominos americanos abarcando 24 países americanos. Esta base de datos georreferenciada puede contribuir no solo a mejorar el conocimiento de las distribuciones geográficas de todas las especies de triatominos americanos, sino también a diseñar mejores estrategias para la promoción de la salud y el control de vectores. Creemos que será de utilidad práctica tanto para la comunidad académica y educativa, como para aquellas instituciones responsables de las actividades de promoción, prevención y control de vectores en Salud Pública.

### Breve resumen de antecedentes de los conjuntos de datos de presencia de especies de triatominos

En 2018 se creó, describió [4.] y almacenó en Figshare [6.] un conjunto de datos inicial (21815 registros georreferenciados) de presencia de especies de triatominos llamado DataTri. Posteriormente, en 2020 se incorporaron 5893 registros (llegando a un total de 27708); luego, fueron modificados al formato Darwin Core [7.] y finalmente se publicó en la plataforma Global Biodiversity Information Facility (GBIF) dividida en dos conjuntos de datos complementarios: el Conjunto de datos Americano (con 11791 registros, abarcando un período entre los años 1926 y 2022) y el Conjunto de datos Argentino (con 15917 registros, abarcando un período entre los años 1912 y 2019) [8.] (Figure 1a). Ambos conjuntos de datos son complementarios ya que el Conjunto de datos Americano incluye todas las especies de triatominos americanos excepto las 17 especies de triatominos (a saber, *Panstrongylus geniculatus* (Latreille, 1811), *P. guentheri* Berg, 1879, *P. megistus* (Burmeister, 1835), *P. rufotuberculatus* (Champion, 1899), *Psammolestes coreodes* Bergroth, 1911, *Triatoma breyeri* Del Ponte, 1929, *T. delpontei* Romaña & Abalos, 1947, *T. eratyrusiformis* Del Ponte, 1929, *T. garciabesi* Carcavallo, Cichero, Martínez, Prosen & Ronderos, 1967, *T. guasayana* Wygodzinsky & Abalos, 1949, *T. infestans* (Klug, 1834), *T. limai* Del Ponte, 1929, *T. patagonica* Del Ponte, 1929, *T. platensis* Neiva, 1913, *T. rubrofasciata* (De Geer, 1773), *T. rubrovaria* (Blanchard, 1843) y *T. sordida* (Stål, 1859)) que se incluyen en el Conjunto de datos Argentino y que tienen (o han tenido) al menos parte de su distribución geográfica dentro del territorio Argentino.

**Figura 1.** Resumen esquemático de la conformación de los conjuntos de datos de triatominos en un marco temporal. N= número de registros en cada conjunto de datos/subconjunto de datos. a) Acciones realizadas entre 2018-2020 y b) acciones realizadas en 2022. La flecha negra indica la eliminación de registros incorrectos (registros repetidos). La flecha azul indica la adición de nuevos registros.

### Descripción de los nuevos datos de ocurrencia de triatominos americanos

Para construir el nuevo subconjunto de datos de ocurrencias de triatominos americano (Subconjunto de datos Americano) e integrarlo en el Nuevo conjunto de datos Americano disponible actualmente, se compilaron un total de 7852 datos de ocurrencia a lo largo de dos años (2020–2022) de revisiones sistemáticas (Figure 1b, Tabla 1). Los nuevos registros (*N* =7852) se identifican consecutivamente desde el “catalogNumber = 27631” al “catalogNumber = 35482” en el Nuevo conjunto de datos americano. Las incorporaciones más importantes del Subconjunto de datos Americano son: (i) la incorporación de nuevas especies de triatominos descritas entre 2018-2021, así como especies (actuales y extintas) no incluidas originalmente en el ‘Conjunto de datos DataTri’, (ii) sinonimias y transferencias formales de especies, y (iii) nuevos registros que actualizan varias presencias temporales y geográficas de especies.

**Tabla 1:** Clasificación taxonómica actual de las especies de triatominos americanos existentes y extintas (†) según la última clasificación taxonómica de Alevi *et al.* [2.]. La “x” indica las especies de triatominos incluidas en el Nuevo conjunto de datos Americano o en el Conjunto de datos Argentino.

Tribus	Géneros	Nombre de las especies (nombre del descriptor/es, año)	Especies incluidas en el Nuevo conjunto de datos Americano	Especies incluidas en el Conjunto de datos Argentino	Número de registros en el Nuevo conjunto de datos Americano	Número de registros contribuidos por el Subconjunto de datos Americano
Alberproseniini	*Alberprosenia*	*goyovargasi* (Martínez & Carcavallo, 1977)	×	-	1	1
		*malheiroi* (Serra, Atzingen & Serra, 1980)	×	-	3	0
Bolboderini	*Belminus*	*corredori* (Galvão & Angulo, 2006)	×	-	1	0
		*costaricensis* (Herrer, Lent & Wygodzinsky, 1954)	×	-	2	0
		*ferroae* (Sandoval, Pabón, Jurberg & Galvão, 2007)	×	-	4	0
		*herreri* (Lent & Wygodzinsky, 1979)	×	-	22	6
		*laportei* (Lent, Jurberg & Carcavallo, 1995)	×	-	3	1
		*peruvianus* (Herrer, Lent & Wygodzinsky, 1954)	×	-	6	0
		*pittieri* (Osuna & Ayala, 1993)	×	-	1	0
		*rugulosus* (Stål, 1859)	×	-	2	1
		*santosmalletae* (Dale, Justi & Galvão, 2021)	×	-	0	1
	*Bolbodera*	*scabrosa* (Valdés, 1910)	×	-	0	3
	*Microtriatoma*	*borbai* (Lent & Wygodzinsky, 1979)	×	-	5	1
		*trinidadensis* (Lent, 1951)	×	-	12	1
	*Parabelminus*	*carioca* (Lent, 1943)	×	-	1	0
		*yurupucu* (Lent & Wygodzinsky, 1979)	×	-	2	0
Cavernicolini	*Cavernicola*	*lenti* (Barrett & Arias, 1985)	×	-	3	0
		*pilosa* (Barber, 1937)	×	-	39	3
Rhodniini	*Psammolestes*	*arthuri* (Pinto, 1926)	×	-	15	6
		*coreodes* (Bergroth, 1911)	-	×	0	0
		*tertius* (Lent & Jurberg, 1965)	×	-	91	17
	*Rhodnius*	*amazonicus* (Almeida, Santos & Sposina, 1973)	×	-	6	2
		*barretti* (Abad-Franch, Pavan, Jaramillo, Palomeque, Dale, Chaverra & Monteiro, 2013)	×	-	2	1
		*brethesi* (Matta, 1919)	×	-	12	7
		*colombiensis* (Mejia, Galvão & Jurberg, 1999)	×	-	23	1
		*dalessandroi* (Carcavallo & Barreto, 1976)	×	-	2	0
		*domesticus* (Neiva & Pinto, 1923)	×	-	17	2
		*ecuadoriensis* (Lent & León, 1958)	×	-	44	64
		*marabaensis* (dos Santos Souza, Barbosa Von Atzingen, Furtado, de Oliveira, Damieli Nascimento, Pagotto Vendrami & Aristeu da Rosa, 2016)	×	-	1	0
		*micki* (Zhao, Galvão & Cai, 2021)	×	-	0	2
		*milesi* (Carcavallo, Rocha, Galvão & Jurberg, 2001 (in: Valente et al. 2001))	×	-	2	1
		*montenegrensis* (Rosa, Rocha, Gardim, Pinto, Mendonça, Ferreira Filho, Carvalho, Camargo, Oliveira, Nascimento, Cilense & Almeida, 2012)	×	-	3	5
		*nasutus* (Stål, 1859)	×	-	66	2
		*neglectus* (Lent, 1954)	×	-	139	9
		*neivai* (Lent, 1953)	×	-	6	1
		*pallescens* (Barber, 1932)	×	-	126	41
		*paraensis* (Sherlock, Guitton & Miles, 1977)	×	-	7	2
		*pictipes* (Stål, 1872)	×	-	164	16
		*prolixus* (Stål, 1859)	×	-	1214	16
		*robustus* (Larrousse, 1927)	×	-	115	6
		*stali* (Lent, Jurberg & Galvão, 1993)	×	-	10	2
		*zeledoni* (Jurberg, Rocha & Galvão, 2009)	×	-	1	0
Triatomini	*Dipetalogaster*	*maxima* (Uhler, 1894)	×	-	15	28
	*Eratyrus*	*cuspidatus* (Stål, 1859)	×	-	59	45
		*mucronatus* (Stål, 1859)	×	-	91	14
	*Hermanlentia*	*matsunoi* (Fernández-Loayza, 1989)	×	-	4	0
	*Mepraia*	*gajardoi* (Frias, Henry & Gonzalez, 1998)	×	-	11	17
		*parapatrica* (Frias-Lasserre, 2010)	×	-	6	6
		*spinolai* (Porter, 1934)	×	-	24	152
	*Nesotriatoma*	*confusa* (de Oliveira, Ayala, Justi, da Rosa & Galvão, 2018)	×	-	2	11
		*flavida* (Neiva, 1911)	×	-	7	1
		*obscura* (Maldonado & Farr, 1962)	×	-	0	3
	*Panstrongylus*	*chinai* (Del Ponte, 1929)	×	-	64	16
		*diasi* (Pinto & Lent, 1946)	×	-	24	8
		*geniculatus* (Latreille, 1811)	-	×	0	0
		*guentheri* (Berg, 1879)	-	×	0	0
		*howardi* (Neiva, 1911)	×	-	3	6
		*humeralis* (Usinger, 1939)	×	-	9	0
		*lenti* (Galvão & Palma, 1968)	×	-	2	0
		*lignarius* (Walker, 1873)	×	-	53	9
		*lutzi* (Neiva & Pinto, 1923)	×	-	614	41
		*martinezorum* (Ayala, 2009)	×	-	3	0
		*megistus* (Burmeister, 1835)	-	×	0	0
		*mitarakaensis* (Bérenger & Blanchet, 2007)	×	-	1	0
		*rufotuberculatus* (Champion, 1899)	-	×	0	0
		*tupynambai* (Lent, 1942)	×	-	16	1
		*hispaniolae* (Ponair Jr., 2013) †	×	-	0	1
	*Paratriatoma*	*hirsuta* (Barber, 1938)	×	-	58	3
		*lecticularia* (Stål, 1859)	×	-	30	200
	*Triatoma*	*amicitiae* (Lent, 1951)	-	-	0	0
		*arthurneivai* (Lent & Martins, 1940)	×	-	8	1
		*bahiensis* (Sherlock & Serafim, 1967)	×	-	1	2
		*baratai* (Carcavallo & Jurberg, 2000)	×	-	11	5
		*barberi* (Usinger, 1939)	×	-	566	32
		*bassolsae* (Alejandre Aguilar, Nogueda Torres, Cortéz Jimenez, Jurberg, Galvão & Carcavallo, 1999)	×	-	2	1
		*bolivari* (Carcavallo, Martínez & Pelaez, 1987)	×	-	11	2
		*boliviana* (Martínez Avendaño, Chávez Espada,Gil, Aranda Asturizaga, Vargas Mamani & Vidaurre Prieto, 2007)	×	-	1	1
		*bouvieri* (Larrousse, 1924)	-	-	0	0
		*brailovskyi* (Martínez, Carcavallo & Pelaez, 1984)	×	-	16	0
		*brasiliensis* (Neiva, 1911)	×	-	1042	77
		*breyeri* (Del Ponte, 1929)	-	×	0	0
		*carcavalloi* (Jurberg, Rocha & Lent, 1998)	×	-	15	1
		*carrioni* (Larrousse, 1926)	×	-	18	20
		*cavernicola* (Else & Cheong, 1977)	-	-	0	0
		*circummaculata* (Stål, 1859)	×	-	29	8
		*costalimai* (Verano & Galvão, 1958)	×	-	44	2
		*deaneorum* (Galvão, Souza & Lima, 1967)	×	-	3	0
		*delpontei* (Romaña & Abalos, 1947)	-	×	0	0
		*dimidiata* (Latreille, 1811)	×	-	2102	549
		*dispar* (Lent, 1950)	×	-	20	245
		*eratyrusiformis* (Del Ponte, 1929)	-	×	0	0
		*garciabesi* (Carcavallo, Cichero, Martínez, Prosen & Ronderos, 1967)	-	×	0	0
		*gerstaeckeri* (Stål, 1859)	×	-	388	3740
		*gomeznunezi* (Martínez, Carcavallo & Jurberg, 1994)	×	-	2	0
		*guasayana* (Wygodzinsky & Abalos, 1949)	-	×	0	0
		*guazu* (Lent & Wygodzinsky, 1979)	×	-	5	4
		*hegneri* (Mazzotti, 1940)	×	-	9	2
		*huehuetenanguensis* (Lima-Cordón, Monroy, Stevens, Rodas, Rodas, Dorn & Justi, 2019)	×	-	10	39
		*incrassata* (Usinger, 1939)	×	-	4	0
		*indictiva* (Neiva, 1912)	×	-	5	192
		*infestans* (Klug, 1834)	-	×	0	0
		*jatai* (Gonçalves, Teves-Neves, Santos-Mallet, Carbajal de la Fuente, Lopes, 2013)	×	-	2	2
		*juazeirensis* (Costa y Felix, 2007)	×	-	17	5
		*jurbergi* (Carcavallo, Galvão & Lent, 1998)	×	-	8	2
		*klugi* (Carcavallo, Jurberg, Lent & Galvão, 2001)	×	-	3	3
		*lenti* (Sherlock & Serafim, 1967)	×	-	16	9
		*leopoldi* (Schoudeten, 1933)	-	-	0	0
		*limai* (Del Ponte, 1929)	-	×	0	0
		*longipennis* (Usinger, 1939)	×	-	381	3
		*maculata* (Erichson, 1848)	×	-	78	96
		*matogrossensis* (Leite & Barbosa, 1953)	×	-	11	5
		*mazzottii* (Usinger, 1941)	×	-	121	30
		*melanica* (Neiva & Lent, 1941)	×	-	22	2
		*melanocephala* (Neiva & Pinto, 1923)	×	-	93	43
		*mexicana (Herrich-Schaeffer, 1848)*	×	-	582	36
		*migrans* (Breddin, 1903)	-	-	0	0
		*mopan* (Dorn, Justi, Dale, Stevens, Galvão, Lima-Cordón & Carlota Monroy, 2018)	×	-	0	7
		*neotomae* (Neiva, 1911)	×	-	10	4
		*nigromaculata* (Stål, 1872)	×	-	10	5
		*nitida* (Usinger, 1939)	×	-	50	27
		*oliveirai* (Neiva, Pinto & Lent, 1939)	×	-	7	1
		*pallidipennis* (Stål, 1872)	×	-	320	8
		*patagonica* (Del Ponte, 1929)	-	×	0	0
		*peninsularis* (Usinger, 1940)	×	-	8	0
		*petrochiae* (Pinto & Barreto, 1925)	×	-	28	4
		*phyllosoma* (Burmeister, 1835)	×	-	38	26
		*picturata* (Usinger, 1939)	×	-	27	0
		*pintodiasi* (Jurberg, Cunha, Cailleaux, Raigorodschi, Souza Lima, da Silva Roch, Figueiredo Moreira, 2013)	×	-	1	0
		*platensis* (Neiva, 1913)	-	×	0	0
		*protracta* (Uhler, 1894)	×	-	341	207
		*pseudomaculata* (Corrêa & Espínola, 1964)	×	-	1392	24
		*pugasi* (Lent, 1953)	-	-	0	0
		*recurva* (Stål, 1868)	×	-	31	12
		*rosai* (Alevi, de Oliveira, Caris Garcia, Cesaretto Cristal, Grzyb Delgado, de Freitas Bittinelli,Visinho dos Reis, Ravazi, Bortolozo de Oliveira, Galvão, Vilela de Azeredo-Oliveira & Fernandez Madeira, 2020)	-	-	0	0
		*rubida* (Uhler, 1894)	×	-	158	515
		*rubrofasciata* (De Geer, 1773)	-	×	0	0
		*rubrovaria* (Blanchard, 1843)	-	×	0	0
		*ryckmani* (Zeledón & Ponce, 1972)	×	-	3	3
		*sanguisuga* (Leconte, 1855)	×	-	124	1006
		*sherlocki* (Papa, Jurberg, Carcavallo, Cerqueira & Barata, 2002)	×	-	13	8
		*sinaloensis* (Ryckman, 1962)	×	-	5	1
		*sinica* (Hsiao, 1965)	-	-	0	0
		*sordida* (Stål, 1859)	-	×	0	0
		*tibiamaculata* (Pinto, 1926)	×	-	69	19
		*vandae* (Carcavallo, Jurberg, Rocha, Galvão, Noireau & Lent, 2002)	×	-	4	6
		*venosa* (Stål, 1872)	×	-	50	12
		*vitticeps* (Stål, 1859)	×	-	114	2
		*williami* (Galvão, Souza & Lima, 1965)	×	-	22	10
		*wygodzinskyi* (Lent, 1951)	×	-	8	2
		*dominicana* (Poinar, 2005) †	×	-	0	1
	*Linshcosteus*	*carnifex* (Distant, 1904)	-	-	0	0
		*chota* (Lent & Wygodzinsky, 1979)	-	-	0	0
		*confumus* (Ghauri, 1976)	-	-	0	0
		*costalis* (Ghauri, 1976)	-	-	0	0
		*kali* (Lent & Wygodzinsky, 1979)	-	-	0	0
		*karupus* (Galvão, Patterson, Rocha & Jurberg, 2002)	-	-	0	0
	*Paleotriatoma*	*metaxytaxa* (Poinar, 2019) †	-	-	0	0
Número total de registros					11748	7852

Se incorporaron siete nuevas especies actuales: *Bolbodera scabrosa* (Valdés, 1910), *Belminus santosmalletae* (Dale, Justi & Galvão, 2021), *Rhodnius micki* (Zhao, Galvão & Cai, 2021), *Nesotriatoma obscura* (Maldonado & Farr, 1962), *Nesotriatoma confusa* (de Oliveira, Ayala, Justi, da Rosa & Galvão, 2018), *Triatoma huehuetenanguensis* (Lima-Cordón, Monroy, Stevens, Rodas, Rodas, Dorn & Justi, 2019) y *T. mopan* (Dorn, Justi, Dale, Stevens, Galvão, Lima-Cordón & Carlota Monroy, 2018), y además se incorporaron dos especies extintas: *Triatoma dominicana* (Poinar, 2005) † y *Panstrongylus hispaniolae* (Ponair Jr., 2013) †. Con estas incorporaciones, el Nuevo conjunto de datos Americano incluye 125 especies que, junto con las 17 especies incluidas en el Conjunto de datos Argentino, suman un total de 142 especies de triatominos. A excepción del caso particular de la especie *T. rosai (Alevi, de Oliveira, Caris Garcia, Cesaretto Cristal, Grzyb Delgado, de Freitas Bittinelli,Visinho dos Reis, Ravazi, Bortolozo de Oliveira, Galvão, Vilela de Azeredo-Oliveira & Fernandez Madeira, 2020)*, que fue descrita en 2020 después de que la última actualización del Conjunto de datos Argentino fuese hecha (y por lo tanto no incluida en este último), los registros de ocurrencia de todas las especies de triatominos Americanos descritas hasta la fecha están incluídos entre ambos conjuntos de datos. (Tabla 1).

Las actualizaciones taxonómicas fueron (i) sinonimias: *Rhodnius taquarussuensis*, que se describió a través de la taxonomía integrativa [9.] y luego fue sinonimizada con *R. neglectus* (Lent, 1954) [10.]; algunos registros de *Nesotriatoma bruneri* fueron sinonimizados con *Nesotriatoma flavida* (Neiva, 1911) [11.]; algunos registros de *Triatoma dimidiata* de Guatemala, Honduras y México fueron sinonimizados con *T. huehuetenanguensis* [12.]; y (ii) transferencias formales: *Triatoma flavida* se transfirió al género Nesotriatoma con la nueva combinación resultante, *Nesotriatoma flavida* [11.] y *Triatoma lecticularia* se transfirió al género *Paratriatoma* con la nueva combinación resultante *Paratriatoma lecticularia* (Stål, 1859) [13.].

La incorporación de nuevos registros del Subconjunto de datos Americano aporta nueva información (respecto al ‘Conjunto de datos DataTri’) y/o actualiza los patrones geográficos de algunas especies y/o subespecies para algunos países. Estos son los casos de nuevas ocurrencias de *Triatoma mopan* y *T. huehuetenanguensis* en Belice [14.–16.] o *T. dispar* (Lent, 1950), *T. nitida* (Usinger, 1939), *T. ryckmani* (Zeledón & Ponce, 1972), *Cavernicola pilosa* (Barber, 1937), *Eratyrus cuspidatus* (Stål, 1859), *Belminus costaricensis* (Herrer, Lent & Wygodzinsky, 1954) y *Microtriatoma trinidadensis* (Lent, 1951) en Costa Rica [17.]. Otros casos para resaltar son el de *Triatoma sanguisuga* (Leconte, 1855) en Nebraska, Estados Unidos [18.], y *T. protracta woodi* (Uhler, 1894), que hasta ahora solo habían sido registrados en el ámbito silvestre [19.] y ahora han sido registrados en hábitats domiciliarios de un condado de Texas [20.] y en peridomicilios del condado de Nuevo México [21.], y *T. rubida uhleri* (Uhler, 1894), que tiene registros en hábitats domiciliarios y un registro en hábitat silvestre [22.]. Con respecto a Guayana Francesa, se agregaron registros de *Belminus laportei* (Lent, Jurberg & Carcavallo, 1995) [23.] aumentando a 11 el número de especies representadas para este país. México es otro ejemplo, con registros de *T. rubida sonoriana* que representan nuevas inclusiones para todos los tipos de hábitat (domicilio, peridomicilio y silvestre) [24.], nuevos registros de *T. barberi* (Usinger, 1939) para el estado de México [25.], así como registros de *T. rubida* para el municipio de Ocampo y registros de *T. gerstaeckeri* (Stål, 1859) [26.]. Se actualizaron además registros de algunas especies en nuevos estados de Brasil, como el caso de *Eratyrus mucronatus* (Stål, 1859) y *Rhodnius montenegrensis* (Rosa, Rocha, Gardim, Pinto, Mendonça, Ferreira Filho, Carvalho, Camargo, Oliveira, Nascimento, Cilense, Almeida, 2012) en Roraima [27.], como así también la presencia de esta segunda especie en el estado de Amazonas [28.]. Por otra parte, se incorporaron nuevas ocurrencias de *Microtriatoma borbai* (Lent & Wygodzinsky, 1979) en Espírito Santo [29.], *Psammolestes tertius* (Lent & Jurberg, 1965) en los estados de Sergipe [30.], Paraná [31.] y Rio Grande do Norte [32.], y de *Cavernicola pilosa* y *Microtriatoma trinidadensis* en Amazonas [33., 34.].

Respecto a la lista de países del Nuevo conjunto de datos Americano, se incluye a Jamaica como nuevo país incorporando registros de la especie *Nesotriatoma obscura* [35.]. A modo de resumen, podemos mencionar que más del 70% de las especies que estaban presentes en el Conjunto de datos Americano tienen ahora nuevos registros incorporados en el Nuevo conjunto de datos Americano (Ver Tabla 1 para más información).

Respecto a los patrones temporales se puede mencionar que la nueva información incluida en el Subconjunto de datos Americano abarca el período desde el año 1935 al año 2022. El 90% de los registros posee información de las fechas, mientras que el 30% de ellos comprende datos de los últimos cuatro años. En cuanto a las actualizaciones de los patrones temporales de algunas especies, un caso destacable son los nuevos registros de ocurrencia de *Rhodnius pictipes* (Stål, 1872) que actualizan su distribución en Trinidad y Tobago desde 1985 [36.].

Como resumen general, la adición de nuevos registros (N= 7852) en el Nuevo conjunto de datos Americano (llegando a los 19600 registros), más los registros incluidos en el Conjunto de datos Argentino (N= 15917) alcanza un total de 35517 registros de ocurrencias para 142 especies de triatominos americanos (Figura 2).

**Figura 2.** Mapa interactivo de las presencias georreferenciadas (Nuevo conjunto de datos Americano) alojadas en GBIF [5.]. https://www.gbif.org/dataset/eae731a7-3e82-4295-b0b3-ec72d75a402d

## Métodos

A continuación, se describen los métodos aplicados para recopilar, georreferenciar y validar las ocurrencias de especies de triatominos del Subconjunto de datos Americano.

### Tipos de fuentes de información y compilación de datos de especies de triatominos

Para construir el Subconjunto de datos Americano, los datos para cada especie de triatomino se obtuvieron mediante una revisión detallada y exhaustiva de la información. No se establecieron límites específicos de rango temporal, para así obtener la mayor cantidad posible de nuevos datos de todos los países Americanos. En cuanto a la información publicada, se revisaron varios tipos de repositorios bibliográficos públicos disponibles en línea (BioOne, Google Scholar, PLoS, PubMed, Scielo, ScienceDirect, Wiley) utilizando términos como “Chagas disease”, “Triatominae”, “*Trypanosoma cruzi*”, sin restricción de lenguaje. También revisamos la base de datos bibliográfica de triatominos, pública y de libre acceso, llamada BibTri [37.]. Cuando los artículos publicados mencionaron conjuntos de datos no publicados, contactamos a los autores y les pedimos que si nos podían proporcionar las coordenadas geográficas o al menos los datos de las localidades para poder georreferenciarlos.

### Proceso de georreferenciación de datos

Para asociar rigurosamente cada registro a una ubicación específica en el espacio geográfico, los datos deben tener información expresada en coordenadas geográficas (latitud y longitud). Si no se disponía de coordenadas geográficas, se utilizaba el nombre del sitio junto con la información de las divisiones administrativas para lograr una ubicación precisa mediante Google Earth [38.]. Si las coordenadas geográficas no se encontraban expresadas en grados decimales, se convirtieron utilizando una aplicación de conversión de coordenadas [39.]. Cuando solo estaban disponibles las coordenadas geográficas, las divisiones administrativas correspondientes se completaron utilizando una herramienta llamada GeoLoc [40.]. El datum (sistema de coordenadas y conjunto de puntos de referencia utilizados para ubicar lugares en la Tierra) utilizado para todos los registros geográficos fue WGS84 (World Geodetic System 1984). El conjunto de datos final se construyó después de un control de calidad de datos (consulte la sección Validación de datos y control de calidad).

### Descripción de los campos del Subconjunto de datos Americano

Se recopiló toda la información relevante y disponible asociada a cada especie de triatomino y adjuntamos los datos a cada campo del conjunto de datos, incluyendo las características de los especímenes recolectados y de los sitios muestreados. Para describir mejor los campos (basados en los términos de Darwin Core [7.]) utilizados para sistematizar la información, los mismos se agruparon en las siguientes seis categorías: 1) identificadores (incluye los campos utilizados para identificar cada registro, e.j. ID de ocurrencia, código de la Institución, idioma del recurso, referencias asociadas, etc.), 2) sistemática (incluye los campos utilizados para la información sistemática, e.j. nombre científico, autoría del nombre científico, rango taxonómico y comentarios taxonómicos), 3) geográfica (incluye los campos con información de divisiones administrativas, coordenadas, fuentes de georreferencia, etc.), 4) temporal (incluye los campos relacionados con la fecha del evento, como año, mes y día), 5) muestreo (incluye los campos relacionados con el proceso de muestreo, como el/los nombre/s del recolector/es de especímenes, el hábitat muestreado, el protocolo y el esfuerzo de muestreo, etc.) y 6) individuo (incluye los campos relacionados con el número total, sexo y etapa de vida de los individuos muestreados). Cada campo independiente forma parte del archivo de datos mencionado en la sección Declaración de Disponibilidad de Datos.

Las siguientes subsecciones proporcionan algunos detalles sobre algunos de los campos mencionados anteriormente que requieren aclaraciones específicas.

#### Campos sistemáticos

Cuando correspondía, el campo ‘taxonRemarks’ incluyó notas y/o referencias sobre sinonimias o transferencias formales de las especies descritas en el registro correspondiente.

#### Campos geográficos

El campo ‘locality’ refiere al nombre del sitio más cercano a las coordenadas geográficas, no necesariamente al nombre de la localidad donde se recolectaron los especímenes. En el caso de que la información geográfica más exacta del registro haya sido el nombre del municipio, las coordenadas corresponden a su centroide.

#### Campos temporales

Cuando la información de un grupo de especímenes correspondía a un período de tiempo determinado, pero con fechas específicas disponibles, los datos se dividieron en diferentes registros. Si dicha división no era posible realizarla, cada registro incluyó la información original del intervalo de tiempo (en años, meses o días) en el campo ‘eventDate’ (e.j., 2005/2006).

#### Campos de muestreo

El campo ‘habitat’ refiere al tipo de hábitat donde se recolectaron los triatominos, clasificados en tres categorías: domicilio, peridomicilio y silvestre. Cuando la información específica del hábitat no podia desagregarse, el hábitat se expresó como una combinación de dos o tres de esas categorías (e.j., domicilio–peridomicilio, domicilio–silvestre, peridomicilio–silvestre o domicilio–peridomicilio–silvestre).

En el campo ‘samplingProtocols’ la información disponible se clasificó en dos categorías principales: (i) búsqueda activa, cuando la búsqueda involucró personal especializado, y (iii) recolección pasiva, cuando se utilizaron diferentes tipos de trampas (e.j., trampas de luz o trampas Noireau).

## Validación de los datos y control de calidad

El Subconjunto de datos Americano fue sometido a un exhaustivo control de calidad. En primer lugar, cada dato fue extraído por una persona y verificado por otras dos personas para garantizar la precisión y verificar que los registros no estuvieran duplicados. Posteriormente, se revisaron los datos para evitar cualquier tipo de error (e.j., tipeo, georreferenciación, ubicaciones incorrectas, sinonimias, errores en la ortografía de las divisiones administrativas, etc.) que pudiera haber ocurrido durante la compilación o el ingreso de datos. Para corregir y eliminar errores tipográficos y ortográficos en los nombres de las divisiones administrativas, utilizamos el software OpenRefine (RRID:SCR_021305) [41.] que ayuda en la detección de este tipo de errores en grandes conjuntos de datos.

Todas las coordenadas geográficas se verificaron utilizando el software SIG de acceso libre (QGIS, RRID:SCR_018507 [42.]) para detectar errores de georreferenciación y ubicaciones incorrectas, asegurando que cada punto correspondiera a una ubicación en el continente y en el país correcto. Cualquier coordenada atípica que detectamos que estuviera geográficamente distante de la distribución conocida de una especie determinada fue investigada para garantizar que fuera correcta. Durante la validación de las coordenadas geográficas, detectamos que algunos datos de ocurrencia estaban ubicados fuera del continente o dentro de cuerpos de agua continentales. Estos datos pueden haber sido georeferenciados erróneamente por los autores de la publicación científica original; sin embargo, cuando consideramos que estos datos eran lo suficientemente valiosos como para incorporarlos, llevamos a cabo el siguiente procedimiento: si los campos ‘country’, ‘stateProvince’, ‘municipality’ y ‘locality’ fueron proporcionados por los autores, asignamos la coordenada geográfica correcta, como se explica en el apartado Proceso de georreferenciación de datos, tomando como referencia el nombre de la localidad aportado por los autores. Para detectar errores de sinónimos taxonómicos, utilizamos la revisión más actualizada de los nombres válidos de las especies de triatominos [2.]. Si se sospechaba que el nombre de alguna especie estaba desactualizado, consultamos la bibliografía actual o solicitamos la opinión experta de colegas.

Por último, mejoramos la calidad de nuestro conjunto de datos final utilizando el validador de datos de GBIF [43.] para identificar y abordar posibles problemas antes de la publicación del conjunto de datos a través del Integrated Publishing Toolkit (IPT, [44.]).

## Potencial de reutilización

Dado que la información de los conjuntos de datos se ha recopilado mediante diferentes procedimientos, esta compilación puede contener algunos sesgos inherentes que deben abordarse cuando se pretenda utilizar los datos. La mayoría de los datos se obtuvieron de artículos publicados en revistas científicas, acompañados por aquellos proporcionados por colegas. Aunque los datos abarcan 25 países, hubo algunos países como Estados Unidos, Costa Rica, Brasil y México para los cuales el volumen de datos fue mayor que para el resto. En el caso de Estados Unidos, el número de datos de ocurrencia incluidos está influenciado por la gran contribución de datos de colegas que lideran un programa científico comunitario de triatominos [45.] y son coautoras de este trabajo (Rachel Curtis-Robles y Sarah A Hamer). Tres notas importantes sobre los datos de ese programa son: (1) como todos los programas de ciencia comunitaria, los registros reflejan lugares y momentos en los que los miembros de la comunidad conocían el programa y optaron por enviar muestras, que pueden no ser completamente representativas de la distribuciones verdaderas de las especies, (2) las fechas reflejan cuándo se recolectó la muestra, pero algunos ejemplares estaban muertos cuando se recolectaron y, por lo tanto, no reflejan la fenología de la especie y (3) algunas muestras ya se han incluido en publicaciones, y se pueden establecer vínculos utilizando el campo ‘otherCatalogNumbers’. En el caso de Costa Rica, pudimos integrar un número importante de registros del Instituto Nacional de Biodiversidad (INBio) y depositados en el Museo Nacional de Costa Rica, proporcionados por uno de los coautores de este trabajo (José Manuel Ayala Landa). Para el caso de México y Brasil, el número de datos de presencia por país incluidos parece estar influenciado principalmente por dos factores: (i) el número de especies de triatominos presentes en cada país (ambos países tienen un gran número de especies de triatominos), y (ii) por la cantidad de datos de ocurrencia publicados y proporcionados por colegas (México y Brasil también son los países con la mayor cantidad de datos recopilados); la explicación para este último factor va más allá del objetivo de este artículo. Con respecto al muestreo de hábitats, reconocemos que existe un sesgo potencial a favor de los hábitats domiciliarios y peridomiciliarios porque son los hábitats de mayor importancia epidemiológica y el objetivo de las campañas de control de vectores. Además, la escasez de datos sobre hábitats silvestres también refiere a la dificultad de los procedimientos de muestreo en la gran variedad de hábitats silvestres utilizados por los triatominos. Finalmente, vale la pena señalar que alrededor del 37% de los registros carecen de información de fecha disponible; por lo tanto, recomendamos que cualquier análisis basado en este conjunto de datos utilice métodos que tengan en cuenta dichos sesgos.

A pesar de los sesgos mencionados anteriormente, el Subconjunto de datos Americano descrito en este documento e integrado en el Nuevo conjunto de datos Americano, más el Conjunto de datos Argentino complementario, constituyen una valiosa compilación de datos geográficos sobre los triatominos Americanos que actualmente es lo más completa, actualizada e integrada. Por lo tanto, todos los conjuntos de datos mencionados tienen una buena representatividad de los datos en cuanto a la cantidad de especies, la cantidad de países y a que cada registro tiene una coordenada geográfica precisa. Además, como estos conjuntos de datos están alojados en un repositorio abierto y público, esperamos que contribuyan a cumplir objetivos nacionales e internacionales como el de promover el intercambio de información biológica, aumentar y mejorar la accesibilidad de dicha información, proporcionar datos biológicos producidos y recopilados en varios países, mejorando el conocimiento tanto de la biodiversidad como de los datos epidemiológicos relacionados con la enfermedad de Chagas.

## Data Availability

El conjunto de “Datos de ocurrencia de triatominos americanos del Laboratorio de Triatominos del CEPAVE (CONICET-UNLP)” ha sido publicado por el Centro de Estudios Parasitológicos y de Vectores (CEPAVE) [46.] y está disponible en el repositorio GBIF bajo exención de dominio público CC0 [5.].

## Notas del editor

Este documento es parte de una serie de artículos de publicación de datos que trabajan con GBIF y cuentan con el apoyo del Programa Especial para la Investigación y Capacitación en Enfermedades Tropicales (TDR), alojado en la Organización Mundial de la Salud [47.].

## Lista de abreviaciones

GBIF: Global Biodiversity Information Facility; IPT: Integrated Publishing Toolkit.

## Aprobación ética

No aplica.

## Consentimiento para Publicación

No aplica.

## Financiamiento

Esta investigación fue financiada por los subsidios PICT N° 2018-1545 (Investigador Principal Dr. Gerardo Marti) de la Agencia Nacional de Promoción Científica y Tecnológica (ANPCyT) y PICT N° 2018-0707 (Investigador Principal Dra. Soledad Ceccarelli).

## Contribución de los autores

SC, AB, MEV, JER y GAM redactaron el protocolo de colecta de datos. SC, AB y GAM solicitaron los datos no publicados a los investigadores. AB y MEV realizaron el control de calidad de todos los datos, RCR y SAH realizan el control de calidad de los datos del programa científico comunitario de triatominos (EE. UU.), y JMAL realizó el control de calidad de los datos del InBio depositados en el Museo Nacional de Costa Rica. SC escribió el primer borrador y todos los autores contribuyeron al manuscrito.

## Información de los autores

SC, AB, MEV, JER y GAM son investigadores de las instituciones estatales argentinas CONICET y CICBA. SC, GAM y AB también son profesores de la Universidad Nacional de La Plata, Argentina. Su investigación se centra en el estudio de la ecoepidemiología y los patrones espaciales de la diversidad de triatominos, con el objetivo de generar pautas para apoyar las decisiones de control de vectores. Estamos especialmente interesados en los patrones de biodiversidad a gran escala de los triatominos del Nuevo Mundo debido a su papel como vectores de la enfermedad de Chagas. S.A.H. es profesora en la Universidad A&M de Texas; RCR es asistente científica de investigación en la Universidad A&M de Texas y también trabaja en una agencia local de control de vectores en California. SAH y RCR trabajan en la ecoepidemiología de la enfermedad de Chagas y los triatominos en el sur de los Estados Unidos, con especial atención a la vida silvestre y los animales domésticos. JMAL es investigador independiente asociado al Museo del Instituto de Zoología Agrícola (MIZA), Maracay, Venezuela.

## References

[1] World Health Organization (WHO). Integrating neglected tropical diseases into global health and development. Fourth WHO Report on neglected Tropical Diseases. World Health Organization, 2017. https://apps.who.int/iris/handle/10665/255011.

[2] AleviKCC, de OliveiraJ, da Silva RochaD Trends in taxonomy of Chagas disease vectors (Hemiptera, Reduviidae, Triatominae): from Linnaean to integrative taxonomy. Pathogens, 2021; 10(12): 1627. doi:10.3390/pathogens10121627.34959582PMC8706908

[3] CarcavalloRU, Curto de CasasSI, SherlockIA Geographical distribution and alti-latitudinal dispersion of Triatominae. In: CarcavalloRU, Galíndez GirónI, JurbergJ, LentH (eds), Atlas of Chagas’ disease vectors in the Americas Vol 3. Editorial. Rio de Janeiro, FIOCRUZ, Brasil: pp. 747–792.

[4] CeccarelliS, BalsalobreA, MedoneP DataTri, a database of American triatomine species occurrence. Sci. Data, 2018; 24(5): 180071. doi:10.1038/sdata.2018.71.PMC591428429688221

[5] CeccarelliS, BalsalobreA, CanoME Datos de ocurrencia de triatominos americanos del Laboratorio de Triatominos del CEPAVE (CONICET-UNLP). Version 1.5. Centro de Estudios Parasitológicos y de Vectores (CEPAVE). Occurrence dataset. 2022; 10.15468/fbywtn.

[6] CeccarelliS, BalsalobreA, MedoneP DataTri, a database of American triatomine species occurrence. Dataset. figshare. 2018; 10.6084/m9.figshare.c.3946936.v1.PMC591428429688221

[7] WieczorekJ, BloomD, GuralnickR Darwin Core: an evolving community-developed biodiversity data standard. PLoS One, 2012; 7(1): e29715. Public Library of Science. doi:10.1371/journal.pone.0029715.22238640PMC3253084

[8] CeccarelliS, BalsalobreA, CanoME Datos de ocurrencia de triatominos presentes en Argentina del Laboratorio de Triatominos del CEPAVE (CONICET-UNLP). Version 1.2. Centro de Estudios Parasitológicos y de Vectores (CEPAVE). Occurrence dataset. 2020; 10.15468/ak8aax.

[9] da RosaJA, JustinoHHG, NascimentoJD A new species of *Rhodnius* from Brazil (Hemiptera, Reduviidae, Triatominae). Zookeys, 2017; 675: 1–25. doi:10.3897/zookeys.675.12024.PMC552388328769676

[10] NascimentoJD, da RosaJA, Salgado-RoaFC Taxonomical over splitting in the *Rhodnius prolixus* (Insecta: Hemiptera: Reduviidae) clade: Are *R. taquarussuensis* (da Rosa *et al.*, 2017) and *R. neglectus* (Lent, 1954) the same species? PLoS One, 2019; 14(2): e0211285. Public Library of Science. doi:10.1371/journal.pone.0211285.30730919PMC6366742

[11] de OliveiraJ, AyalaJM, JustiSA Description of a new species *of Nesotriatoma Usinger*, 1944 from Cuba and revalidation of synonymy between *Nesotriatoma bruneri* (Usinger, 1944) and N. flavida (Neiva, 1911) (Hemiptera, Reduviidae, Triatominae). J. Vector Ecol., 2018; 43(1): 148–157. doi:10.1111/jvec.12294.29757512

[12] Lima-CordónRA, MonroyMC, StevensL Description of *Triatoma huehuetenanguensis* sp. N., a potential chagas disease vector (Hemiptera, Reduviidae, Triatominae). Zookeys, 2019; 820: 51–57. doi:10.3897/zookeys.820.27258.PMC636187630728739

[13] de PaivaVF, de OliveiraJ, GalvãoC Formal assignation of the kissing bug *Triatoma lecticularia* (Hemiptera: Reduviidae: Triatominae) to the genus Paratriatoma. Insects, 2021; 12(6): 538. doi:10.3390/insects12060538.34200543PMC8227924

[14] DornPL, JustiSA, DaleC Description of *Triatoma mopan* sp. N. from a cave in Belize (Hemiptera, Reduviidae, Triatominae). Zookeys, 2018; 775: 69–95. doi:10.3897/zookeys.775.22553.PMC605800430057472

[15] Lima-CordónRA, CahanSH, McCannC Insights from a comprehensive study of *Trypanosoma cruzi*: A new mitochondrial clade restricted to North and Central America and genetic structure of TcI in the region. PLoS Negl. Trop. Dis., 2021; 15(12): e0010043. doi:10.1371/journal.pntd.0010043.34919556PMC8719664

[16] PitaS, LoriteP, CuadradoA High chromosomal mobility of rDNA clusters in holocentric chromosomes of Triatominae, vectors of Chagas disease (Hemiptera-Reduviidae). Med. Veter. Entomol., 2022; 36(1): 66–80. doi:10.1111/mve.12552.34730244

[17] Ayala LandaJM. Los triatominos de Costa Rica (Heteroptera: Reduviidae, Triatominae). Arq. Entomol., 2017; 18: 189–215.

[18] NielsenDH, KochK, RoachellW First record of an established population of *Triatoma sanguisuga* (Hemiptera: Reduviidae) in Richardson County, Nebraska. J. Med. Entomol., 2021; 58(6): 2519–2523. doi:10.1093/jme/tjab122.34283239

[19] WoodSF. Notes on the distribution and habits of reduviid vectors of Chagas’ disease in the southwestern United States (Hemiptera, Reduviidae). Pan-Pac. Entomol., 1941; 17: 85–94.

[20] GorchakovR, TrosclairLP, WozniakEJ *Trypanosoma cruzi* infection prevalence and bloodmeal analysis in Triatomine vectors of Chagas disease from rural peridomestic locations in Texas, 2013–2014. J. Med. Entomol., 2016; 53(4): 911–918. doi:10.1093/jme/tjw040.27106934

[21] WoodSF. Conenose bug and trypanosome observations for 1957 from southwestern national parks and monuments. Bull. South. Calif. Acad. Sci., 1958; 57: 113–114.

[22] WoodSF. Additional bug (Hemiptera, Reduviidae) annoyance and *Trypanosoma cruzi* in southwestern national monuments. Bull. South. Calif. Acad. Sci., 1954; 53: 52–56.

[23] Gil-SantanaHR, de OliveiraJ. First record of *Belminus laportei* Lent, Jurberg & Carcavallo, 1995 (Hemiptera: Reduviidae: Triatominae) from French Guiana. Rev. Chil. Entomol., 2021; 47(3): doi:10.35249/rche.47.3.21.02.

[24] Paredes GonzálezEA, Álvarez HernandezG, Ortega-GarcíaJ. Transmitter insect of Chagas disease in northwest Mexico: a comparative study of the cuticular hydrocarbons profile of three populations of *Triatoma rubida*: Peridomestic, Domestic and Sylvatic. In: VeeranootN, HeliehS (eds), Chagas Disease—Basic Investigations and Challenges. InTech.

[25] RivasN, González-GuzmánS, Alejandre-AguilarR. First record of *Triatoma barberi* Usinger, 1939 (Hemiptera: Reduviidae) in northern state of Mexico, Mexico. J. Vector Ecol., 2018; 43(2): 337–339. doi:10.1111/jvec.12319.30408287

[26] Martinez-TovarJG, Rodriguez-RojasJJ, Arque-ChungaW Nuevos registros geográficos y notas de infección de *Triatoma gerstaeckeri* (Stal) y *Triatoma rubida* (Uhler) (Hemiptera: Reduviidae: Triatominae) en Nuevo León y Coahuila, México. Acta Zool. Mex., 2013; 29: 227–233.

[27] Gama NetoJ de L, de OliveiraJ, da RosaJA Two new records of Triatominae (Hemiptera: Reduviidae) from Roraima state, Brazil. Rev. Chil. Entomol., Soc. Chil. Entomol., 2020; 46(2): 321–327. doi:10.35249/rche.46.2.20.23.

[28] MadeiraFP, MenezesALR, de JesusAC First report of *Rhodnius montenegrensis* (Hemiptera, Reduviidae, Triatominae) in Amazonas, Brazil. Rev. Soc. Bras. Med. Trop., 2020; 53: e20190436. doi:10.1590/0037-8682-0436-2019.32187341PMC7094056

[29] Gil-SantanaHR, dos Santos MartinsD, da SilvaJB First report of *Microtriatoma borbai* Lent & Wygodzinsky, 1979 (Hemiptera, Reduviidae, Triatominae) in the state of Espírito Santo, Brazil: would *M. borbai* be living in eucalyptus crops? Rev. Soc. Bras. Med. Trop. Soc. Bras. Med. Trop., 2021; 54: e01472021. doi:10.1590/0037-8682-0147-2021.PMC818689434105628

[30] de OliveiraJ, da RosaJA, FontesFM *Psammolestes tertius* Lent & Jurberg, 1965 (Hemiptera, Reduviidae, Triatominae): first report in Sergipe State, Brazil. Rev. Soc. Bras. Med. Trop., 2021; 54: e0708-2020. doi:10.1590/0037-8682-0708-2020.33759924PMC8008867

[31] de PaivaVF, da RosaJA, CerettiWJr Confirmation of the first report of *Psammolestes tertius* Lent and Jurberg, 1965 (Hemiptera, Reduviidae, Triatominae) in Paraná state, Brazil. Rev. Soc. Bras. Med. Trop., 2021; 54: e0485-2020. doi:10.1590/0037-8682-0485-2020.33656150PMC8008866

[32] da SilvaANB, DiotaiutiL, da CâmaraACJ First report of *Psammolestes tertius* Lent & Jurberg, 1965 (Hemiptera, Reduviidae, Triatominae) in Rio Grande do Norte State, Brazil. Check List, 2018; 14(6): 1109–1113. doi:10.15560/14.6.1109.

[33] dos Santos SouzaE, Silva MonteGL, de PaivaVF *Microtriatoma trinidadensis* (Lent, 1951) (Hemiptera, Reduviidae, Triatominae): first record in the state of Amazonas, Brazil. Check List, 2019; 15(5): 905–909. doi:10.15560/15.5.905.

[34] NascimentoEB do, Souza É dosS, PaivaVF de *Cavernicola pilosa* Barber, 1937 (Hemiptera, Reduviidae, Triatominae): first record in Amazonas state, Brazil. Check List, 2020; 16(2): 387–390. doi:10.15560/16.2.387.

[35] Maldonado-CaprilesJ, FarrTH. On some Jamaican Triatominae and Emesinae (Reduviidae: Hemiptera). Proc. Entomol. Soc. Wash., 1962; 64(3): 187–194.

[36] HyltonA, FitzpatrickDM, SuepaulR Preliminary characterization of triatomine bug blood meals on the Island of Trinidad reveals opportunistic feeding behavior on both human and animal hosts. Trop. Med. Infect. Dis., 2020; 5(4): 166. doi:10.3390/tropicalmed5040166.33158108PMC7709638

[37] CEPAVE: BibTri. https://bibtri.cepave.edu.ar/webbibtri.php?wbt_view=1&lang=es. Accessed 2022 May 18.

[38] Google: Google Earth. https://www.google.com.ar/intl/es/earth/. Accessed 2022 May 18.

[39] SpeciesLink: Converter. https://splink.cria.org.br/conversor?criaLANG=en. Accessed 2022 May 17.

[40] SpeciesLink: GeoLoc. https://splink.cria.org.br/geoloc?criaLANG=pt. Accessed 2022 May 18.

[41] OpenRefine. https://openrefine.org/. Accessed 2022 May 18.

[42] QGIS Development Team. QGIS Geographic Information System. Open Source Geospatial Foundation Project. https://qgis.org/.

[43] Global Biodiversity Information Facility (GBIF). Data Validator. https://www.gbif.org/tools/data-validator. Accessed 2022 May 18.

[44] Global Biodiversity Information Facility (GBIF). IPT: The Integrated Publishing Toolkit. https://www.gbif.org/en/ipt. Accessed 2022 May 18.

[45] Curtis-RoblesR, WozniakEJ, AucklandLD Combining public health education and disease ecology research: using citizen science to assess chagas disease entomological risk in Texas. PLoS Negl. Trop. Dis., 2015; 9(12): e0004235. doi:10.1371/journal.pntd.0004235.26658425PMC4687635

[46] Datos de ocurrencia de triatominos americanos del Laboratorio de Triatominos del CEPAVE (CONICET-UNLP). CEPAVE. 2022. https://ipt.mincyt.gob.ar/resource?r=triatominos.

[47] Vectors of human disease series. GigaByte. 2022; 10.46471/GIGABYTE_SERIES_0002.
